# There is no privileged link between kinds and essences early in development

**DOI:** 10.1073/pnas.2003627117

**Published:** 2020-05-04

**Authors:** Alexander Noyes, Frank C. Keil

**Affiliations:** ^a^Department of Psychology, Yale University, New Haven, CT 06520

**Keywords:** social cognition, conceptual development, categorization, causal reasoning

## Abstract

According to the dominant view of category representation, people preferentially infer that kinds (richly structured categories) reflect essences. Generic language (“Boys like blue”) often occupies the central role in accounts of the formation of essentialist interpretations—especially in the context of social categories. In a preregistered study (*n* = 240 American children, ages 4 to 9 y), we tested whether children assume essences in the presence of generic language or whether they flexibly assume diverse causal structures. Children learned about a novel social category described with generic statements containing either biological properties or cultural properties. Although generic language always led children to believe that properties were nonaccidental, young children (4 or 5 y) in this sample inferred the nonaccidental structure was socialization. Older children (6 to 9 y) flexibly interpreted the category as essential or socialized depending on the type of properties that generalized. We uncovered early-emerging flexibility and no privileged link between kinds and essences.

Kinds are richly structured (e.g., *tiger*), whereas mere categories are shallow (e.g., *white things*). Consequently, kinds promote induction and explanation (1, 2) and are fundamental to higher-order cognitive processes ranging from scientific reasoning (3) to social cognition (4). Here, we ask how children represent kinds—using social categories as revealing cases.

According to psychological essentialism, people represent richly structured categories as having essences—internal, natural causes responsible for the category and its correlated features (5, 6). For example, people often assume that boys and girls are inherently different (7). Under a contrasting theory, people flexibly infer kinds are either naturally determined or socially constructed (8). Causal structures can create stable kinds without those structures being internal to members, that is, external, social structures. At stake, then, is whether children assume kinds (categories with high inductive potential, nonaccidental features, and generalizable causal structures) are essential (i.e., naturally determined) or whether they also infer alternative causal structures (i.e., social construction).

Generic statements (e.g., “ducks lay eggs” and “boys like blue”) distinguish these theories because generic statements signal kinds (9). Supporting a link between kind representations and essentialism, several studies found that generic statements increased essentialist reasoning in children and adults (9–11). Elsewhere, however, adults inferred that kinds could be naturally determined or socially constructed (8). Adults inferred a novel social category as essential when generic statements contained biological properties and as socially constructed when generic statements contained cultural properties.

Although adults’ kind representations appeared flexible, psychological essentialism may be developmentally primitive. In one study on gender, children believed that physical properties (e.g., “has breasts”) and behavioral properties (e.g., “likes to sew”) were both essential, whereas adults believed only physical properties were essential (12). Therefore, children may fail to distinguish between biological and cultural generics and default to essentialism. If true, this default would indicate a special connection between kinds and essences, such that adults overcome an early-emerging essentialist bias.

To contrast these theories, we conducted a preregistered study (https://osf.io/9tpxa). Two hundred forty children (ages 4 to 9 y) learned about a novel social category described with either biological generics (e.g., “Vawnsies feel sick when they drink milk”) or cultural generics (e.g., “Vawnsies believe that fish talk to God”). We measured kindhood via children’s evaluations of formal explanations; for example, “Look, he has freckles on his feet. Is that because he’s a Vawnsie?” A formal explanation (which uses category membership as the sole basis of explanation) indicates the property is nonaccidentally linked to the category (12, 13). Endorsing multiple formal explanations indicates the category is richly structured. We measured essentialism using a switched-at-birth paradigm (12). Children were told a Vawnsie woman had a baby raised by an American woman. Participants were asked whether Vawnsie properties would be absent or present when the baby was older. Agreement indicates the property is naturally determined; disagreement indicates the property is socially constructed. The primary advance over previous developmental studies (e.g., ref. 11) is that we systematically varied property content (instead of presenting both properties in a single condition), and we measured kindhood separately from essentialism (instead of including measures of kindhood within a single “essentialism” composite measure). Together, these advances allowed us to test whether generic language induced kind representations and whether children were biased to assume that kinds reflect essences (i.e., to assume kinds are naturally determined rather than socially constructed).

We predicted that generic language would increase kindhood and that 1) if children initially do not distinguish between biological and cultural generics they should reveal no bias to infer kinds as reflecting essences and that 2) once children notice the contrast they should have flexible representations of kinds.

## Results

**Fig. 1.**
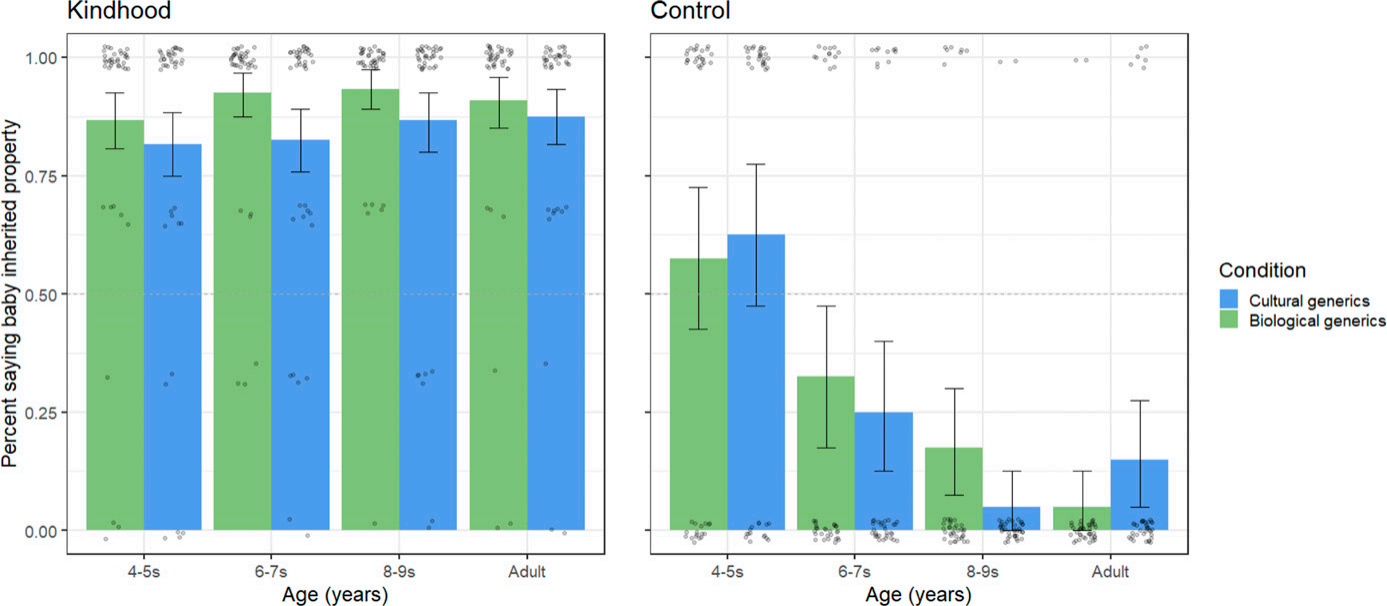
Kindhood and control measure. Children’s endorsement of formal explanations on experimental trials (three trials) compared to the control question (one trial). Midpoint line indicates chance (50%). Error bars are 95% bootstrapped confidence intervals. Age was analyzed continuously but is depicted by group. Dots depict average responses per participant.

Generics increased kindhood (Fig. 1), as indicated by greater endorsement of formal explanations compared to controls: (multilevel logistic regression) *b* = 3.56, SE = 0.56, *P* < 0.001; (McNemar’s χ^2^) χ^2^ (1, 239) = 118.37, *P* < 0.001. Children distinguished between test and control questions more with age (continuous, 4 to 9 y): (multilevel linear regression; logistic regression failed to converge) *b* = 0.15, SE = 02, *P* < 0.001. The widening gap between the test and control reflected changing responses to control questions: (multilevel logistic regression) *b* = −0.75, SE = 0.15, *P* < 0.001, not test questions: *b* = 0.11, SE = 0.60, *P* = 0.860. Critically, the test–control effect held among the youngest children: (McNemar’s χ^2^) χ^2^ (1, 79) = 13.14, *P* < 0.001. All children endorsed formal explanations more often than predicted by chance (87%): (multilevel logistic regression) *b* = 7.49, SE = 0.75, *P* < 0.001. Kindhood was marginally higher when biological generics were used (91%) than when cultural generics were used (84%). The effect was nonsignificant in a multilevel regression: *b* = −0.70, SE = 0.79, *P* = 0.375 and significant in a *t* test: *t* (220.39) = −2.07, *P* = 0.039, *d* = −0.27. Nevertheless, formal explanations were endorsed above chance levels in all conditions and age groups (Fig. 1). Thus, generics succeeded in inducing kind representations.

**Fig. 2.**
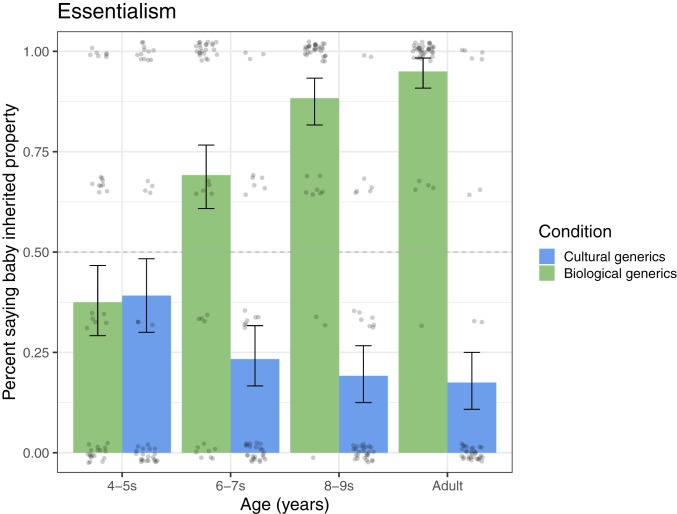
Essentialism measure. Children’s belief that the category is essential (i.e., naturally determined) as indicated by the switched-at-birth paradigm. Children’s report that a baby inherited Vawnsie properties despite being raised in a different cultural context (three total). Midpoint line indicates chance (50%). Error bars are 95% bootstrapped confidence intervals. Age was analyzed continuously but is depicted by group. Dots depict average responses per participant.

Children drew different conclusions about kinds described with biological generics and cultural generics (Fig. 2). Children said the baby inherited properties in the biological condition (65%) more often than in the cultural condition (27%): (multilevel binomial logistic regression [mlm]) *b* = −4.36, SE = 0.84, *P* < 0.001; (*t* test on average) *t* (236.08) = −7.46, *P* < 0.001. This effect was qualified by an interaction with age: *b* = −2.05, SE = 0.38, *P* < 0.001 (Fig. 2).

The youngest children (4 or 5 y) did not distinguish between biological and cultural properties: (mlm) *b* = 0.09, SE = 0.96, *P* = 0.921; (*t* test) *t* (76.54) = 0.18, *P* = 0.861. However, they did not fall back on essentialism. Instead, they tended to assume properties were socialized (38%), overall at below chance levels: (mlm) *b* = −1.36, SE = 0.56, *P* = 0.015; (*t* test) *t* (79) = −2.48, *P* = 0.015.

Robust flexibility emerged by age 6 y (Fig. 2). The 6- to 7-y-old children differentiated between condition: (mlm) *b* = −5.18, SE = 1.67, *P* = 0.002; (*t* test) *t* (74.72) = −5.51, *P* < 0.001. They saw biological generics as indicating natural kinds (69%): (mlm) *b* = 6.71, SE = 2.03, *P* = 0.001; (*t* test) *t* (39) = 2.96, *P* = 0.005 and cultural generics as indicating social kinds (23%): (mlm) *b* = −2.18, SE = 0.70, *P* = 0.002, (*t* test) *t* (39) = −5.10, *P* < 0.001. Older children and adults were the same (Fig. 2).

### Exploratory.

Kindhood did not significantly predict essentialism: *b* = 0.27, SE = 0.51, *P* = 0.591. Instead, there was a significant interaction: *b* = −2.16, SE = 1.01, *P* = 0.033. Children’s tendency to report “yes” to kindhood questions predicted nonsignificantly more essentialist responding in the biological condition (*b* = 1.32, SE = 0.76, *P* = 0.083) and nonsignificantly less essentialist responding in the cultural condition (*b* = −0.87, SE = 0.68, *P* = 0.193).

## General Discussion

Results indicate no privileged link between kinds and essences in childhood. Young children in this sample inferred the kind presented was socialized. By age 6 y, children assumed the kind was inherited when described with biological generics and socialized when described with cultural generics. Thus, children endorsed formal explanations for properties they viewed as extrinsic and social; for example, she has this property because she is a “Vawnsie,” and the property was determined by cultural upbringing. Formal explanations imply the property is linked to the kind’s causal structure. Therefore, children believed kinds could be socially constructed.

In contrast to our findings, previous work finds that preschool-aged children are biased to assume that diverse gender-linked properties are essential. Also, prior theorizing has generics as playing a probable role in transmitting these essentialist beliefs to children. However, children’s tendency to infer that gender is essential should not be generalized to other social categories. Gender may be special (14): Across cultures, children assume gender is natural—potentially indicating either widespread gender-specific input or early-emerging biases in reasoning about gender. Children may hear greater frequencies of generics describing biological properties with respect to gender and greater exposure to apparent physical differences between men and women, which could induce essentialism among younger children. Overcoming this particular assumption is a protracted and context-specific process (14). In contrast, young children do not assume race or novel social categories are essential (14, 15).

Children are flexible across categories. Initially, children see gender and animals as natural kinds and teams and social categories as social kinds. As they improve their updating of beliefs based on generic statements, they learn that single categories can involve multiple causal processes (e.g., the interaction of biology and socialization). Children do not start by privileging essential structure in their representations of kinds.

## Methods

### Participants.

We planned a sample of 40 participants per age group (4 or 5, 6 or 7, and 8 or 9 y and adult) per condition (cultural generic and biological generic). Assuming flexible kind representations produce ∼70% natural responses in the biological condition and 30% in the cultural condition, this provides 95% power to detect a condition difference. This study was approved by Yale Institutional Review Board 1311013027: Cognitive & Metacognitive Development. All participants provided informed consent.

### Design and Procedure.

#### Stimuli.

The novel group wore similar clothing and was diverse in race and gender and ambiguous in age. Children received 16 generic statements in each condition (properties overlapped substantially with refs. 8 and 11), depicted by a single category member each. The biological properties presented at test were “Vawnsies have freckles on their feet,” “…have super strong fingernails,” and “…have breath that smells like maple syrup.” The cultural properties presented at test were “…love eating red flowers,” “…spin in circles when the sun goes down,” and “…think stones can come alive.” We selected low-frequency or unfamiliar properties.

#### Kindhood.

There were three test trials. Children were told an individual has a property. They were then asked whether they had that property “because s/he is a Vawnsie.” A control trial introduced a new property (from the opposite condition) using specific language; this allowed us to test children’s baseline expectations about properties in the absence of generic language.

#### Essentialism.

Switched-at-birth trials assessed the same properties as the formal explanations. Questions focused on absence or presence of Vawnsie properties (contrast to 11, which asked children whether a baby would grow up to have Vawnsie properties or American properties). America was selected as the contrasting culture because children in our sample would know Americans do not exhibit Vawnsie properties.

#### Memory.

Two embedded memory questions were in the prompt. Children performed better than predicted by chance (87% for cultural generics and 92% for biological generics), *P* < 0.001; children’s accuracy did not differ between condition, *P* < 0.926.

### Data Availability.

The complete data file is publicly available at https://osf.io/t9emx/. The original R analysis file is provided for reproducing the analyses (16).
